# Vaccine-Induced Immunological Memory in Invasive Fungal Infections – A Dream so Close yet so Far

**DOI:** 10.3389/fimmu.2021.671068

**Published:** 2021-04-21

**Authors:** Partha S. Biswas

**Affiliations:** Division of Rheumatology & Clinical Immunology, University of Pittsburgh, Pittsburgh, PA, United States

**Keywords:** fungus, invasive infections, immunity, memory response, vaccine

## Abstract

The invasive fungal infections (IFIs) are a major cause of mortality due to infectious disease worldwide. Majority of the IFIs are caused by opportunistic fungi including *Candida*, *Aspergillus* and *Cryptococcus* species. Lack of approved antifungal vaccines and the emergence of antifungal drug-resistant strains pose major constraints in controlling IFIs. A comprehensive understanding of the host immune response is required to develop novel fungal vaccines to prevent death from IFIs. In this review, we have discussed the challenges associated with the development of antifungal vaccines. We mentioned how host-pathogen interactions shape immunological memory and development of long-term protective immunity to IFIs. Furthermore, we underscored the contribution of long-lived innate and adaptive memory cells in protection against IFIs and summarized the current vaccine strategies.

## Introduction

Every year around 1.5 million death occur due to invasive fungal infections (IFIs) worldwide (1). Approximately, 80% of this mortality are due to infections caused by opportunistic fungi (1). Majority of these patients die due to fungal sepsis caused by uncontrolled fungal growth. Additionally, IFIs can impact vital internal organs like lungs, heart, brain, kidneys, and liver leading to end-organ damage. Death due to fungal sepsis and end-organ damage are commonly seen in immunocompromised patients or individuals with inborn errors in immunity, who are unable to control IFIs (2). With the increase in the number of immunocompromised patients, there is an unmet clinical need to develop fungal vaccines to prevent these fatal infections.

One of the major IFIs is invasive Candidiasis or disseminated Candidiasis (2–4). The causative agent for invasive Candidiasis is *Candida* species, more commonly *Candida albicans*, responsible for causing around 46–75% of all IFIs. When *Candida* spp gain access to the bloodstream, it causes significantly high mortality (rates 40-60%), particularly in hospitalized patients and immunocompromised individuals (1, 5). Another cause of IFIs is invasive Aspergillosis, which is caused by a saprophytic bacteria *A. fumigatus*. Approximately, 200,000 people die due to invasive Aspergillosis each year, with mortality rates as high as 30–95% (1). Cryptococcosis, caused by *Cryptococcus neoformans* infects over a million individuals with 20–70% mortality rate annually (1, 6).

It is now well established that occurrence and severity of IFIs are associated to the immune status of the host (7). Majority of the these pathogenic fungi are opportunistic pathogens and cause disease when immune system is suppressed (1). Consequently, the prevalence of IFIs has significantly increased due to alarming rise in the number of HIV and cancer patients and use of immunosuppressive medications. Hence, treating IFIs in the face of compromised immune system is clinically daunting. Thus, to reduce the global incidence and mortality of IFIs, there is a serious need for the development of effective and safe fungal vaccines.

In this review, we systematically discussed the complex interactions between *Candida*, *Aspergillus* and *Cryptococcus* species and the host immune system. We emphasized the role of innate and adaptive immune cells in the context of long-term antifungal memory response and protective immunity. We also outlined the recent efforts in developing candidate antifungal vaccine strategies against IFIs.

## Challenges Associated With the Development of Fungal Vaccines

Majority of the IFIs affect patients with severe immunodeficiency (8). Indeed, patients with compromised immune system and long-term hospitalized patients undergoing invasive medical interventions are at higher risk of developing these systemic infections (1, 8, 9). With the rise in the incidence of cancer and use of immunosuppressive and invasive medical therapies, the incidence and severity of IFIs are likely to increase with time. In the case of invasive Candidiasis, the crude mortality rate can reach as high as 60% in patients with immune deficiency (10). Similarly, *C. neoformans* cause severe meningoencephalitis in HIV patients (11). The mortality rates for invasive Aspergillosis can reach up to 80% in patients with compromised immune system (8, 12, 13).

Treatment with current antifungal drugs have several limitations. These include lack of early diagnostic tools, renal toxicity, development of drug resistance, narrow range of antifungal activity and major drug-drug interactions (14). Thus, there is a demanding clinical need to develop safe and effective antifungal vaccines to mitigate the risk of fatality in these patients. Currently, various research groups have devoted enormous resources to develop effective fungal vaccines. These research efforts are mainly directed against using novel vaccine formulations to generate long-term protective memory in both high-risk patients as well as in healthy individuals (8, 15). However, generation of vaccine-induced protective immunity in immunocompromised subjects is a key clinical challenge (16). Additionally, generating sterile immunity by vaccinating against commensal fungus (e.g., *Candida* spp) could be challenging and in worst case scenario may lead to autoimmunity. The potential high costs in preparing the fungal vaccines to vaccinate a small group of high-risk individuals does not motivate big pharmaceutical companies to develop vaccines against IFIs. Most importantly, designing an effective strategy for vaccination against IFIs is impeded by our lack of understanding of antifungal immunity. Despite these challenges, clinical trials of vaccines against these opportunistic fungi are ongoing and producing encouraging results, which have been described in this review in a systematic manner.

## Characterization of Immunological Memory Response in IFIs

Vaccines trigger short and long-term protective immunity against infections. One of the hallmarks of vaccines is the development of anti-pathogen memory response in the host. The immunological memory against past infections aids in the rapid protection during subsequent assault by the same pathogen. Numerous studies have shown that both innate and adaptive immune cells can memorize previous infections and launch pathogen-specific immunity during secondary infections (17, 18). This is particularly evident in case of many IFIs that cause life threatening diseases. Here, we systematically outline the innate and adaptive immune components responsible for generating innate and adaptive antifungal memory response in mice and human settings.

## Innate Immune Components of Functional Antifungal Memory Response

### Neutrophils

Neutrophils are the first line of defense against infection and play important role in the clearance of the invading pathogens. This process is primarily mediated by two strategies: phagocytosis and secretion of anti-microbials. The role of neutrophils in controlling IFIs is emphasized by the fact that neutropenia is a risk factor for invasive Candidiasis (19, 20) and Aspergillosis (21), but not Cryptococcosis (22). There are multiple ways by which neutrophils control IFIs. The primary and secondary granules of neutrophil contain various antimicrobial peptides, proteolytic and nucleolytic enzymes; all of which are important for the fungicidal activity. Additionally, neutrophils produce reactive oxygen species (ROS) to kill phagocytosed fungi within the phagolysosome (20). Neutrophils facilitate the infiltration of other immune cells to the infection site *via* production of chemokines (23). The neutrophils also inhibit fungal growth through the deprivation of essential nutrients (24). Moreover, neutrophils produce neutrophil extracellular traps (NETs), which are meshwork of extracellular chromatin DNA, histones, and antimicrobial peptides to trap and kill pathogens (24, 25). Notably, the NETs play important role for the killing of the pathogenic fungal hyphae (24, 25).

### Monocytes and Macrophages

Macrophages are scavenger cells, which are derived from blood monocytes. The tissue-resident macrophages reside within the tissues while others remain in the circulation and secondary lymphoid organs and migrate to the sites of infection (26, 27). Upon fungal recognition, macrophages undergo polarization into classical activated macrophages (M1) and alternative activated macrophages (M2) (26, 27). M1 macrophages contribute to fungus clearance by producing pro-inflammatory and fungicidal factors, whereas M2 macrophages support fungal persistence and take part in the resolution of inflammation and tissue healing process (28). On the other hand, tissue-resident macrophages act as a source of pro-inflammatory cytokines and chemokines, which facilitate the migration of other innate and adaptive immune cells to the infected tissue (29).

The classical monocytes, as defined by CD16 and CD14 expression, control fungal infection by inhibiting the conidial germination of *A. fumigatus* and *C. albicans* (30, 31). The monocytes drive the production of IL-1β and prostaglandin E2 production following *C. albicans* infection and facilitate protective T-helper 17 (Th17) differentiation (31). Similar antifungal activity of monocytes were also demonstrated in Cryptococcosis (32).

### Natural Killer (NK) Cells

Unlike neutrophils and macrophages, the role of NK cells in fungal clearance in IFIs is disputed. This is particularly evident in mouse model of invasive Candidiasis, where depletion of NK cells is linked to either no effect or increased susceptibility to *C*. *albicans* in two separate studies, respectively (33, 34). Additionally, NK cells depleted SCID mice succumbed to invasive Candidiasis. However, NK cell depletion had minimal impact on antifungal immunity in animals with normal immune system (35). In sharp contrast, another report demonstrated the contribution NK-cells specific IL-17 receptor signaling in antifungal immunity in invasive Candidiasis (36).

### Dendritic Cells (DCs)

**Figure 1:**
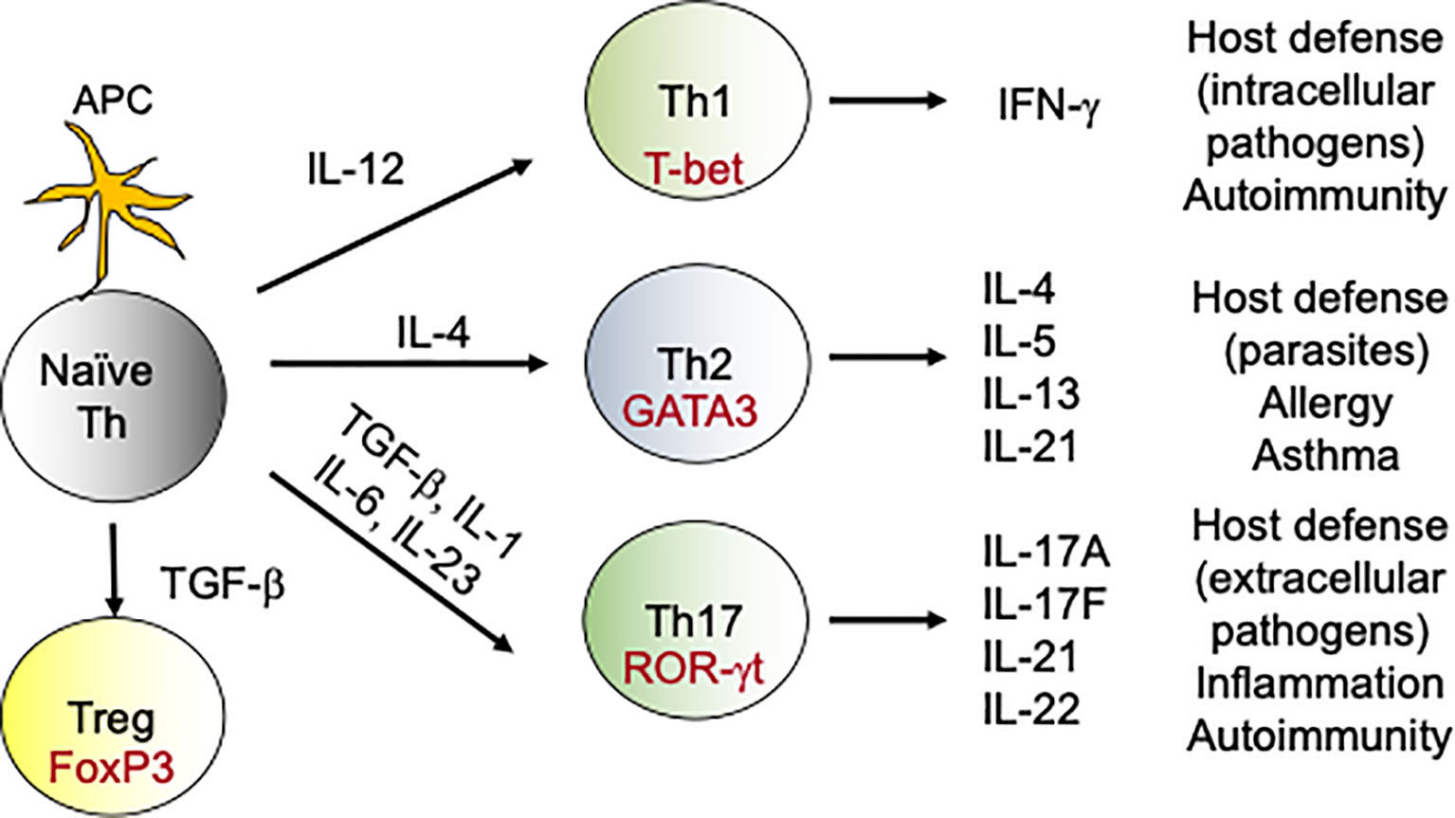
Differentiation of T-helper cells: During fungal infections, DCs present antigens to naive CD4 T cells. During DCs:T cells interaction, DCs produce cytokines which drives the differentiation of various Th subsets. For example, naïve CD4 T cells in the presence of IL-12 upregulate transcription factor T-bet and become Th1 cells. Similarly, IL-6, IL-1 and IL-23 aid in the differentiation of Th17 cells, which produce IL-17 family of cytokines. These subsets (Th1, Th2, Th17 and Tregs) by virtue of their cytokine production exert various effector functions, which modulates the immune response against the fungus. In the context to antifungal immunity, activated Th1 and Th17 cells migrate to the site of infection from secondary lymphoid organs and help in the fungal clearance.

DCs are less efficient in clearing fungal infections in comparison to neutrophils and macrophages (37). Rather, DCs orchestrate the primary immune response by acting as professional antigen presenting cells (APCs) (37). After fungal recognition, DCs process the exogenous fungal antigens and present it to the naïve T cells in context of major histocompatability complex II (MHC II). Additionally, DCs produce cytokines that aid in the differentiation of naïve CD4 T cells towards various T-helper (Th) subsets including Th1, Th2 and Th17 and T regulatory (T regs) cells (38) (**Figure 1**). Interestingly, DCs direct different Th subsets development based on the fungal recognition of yeast and hyphal forms of *C. albicans* (39). In addition to its role as APCs, DCs have been shown to protect mice and human from *A*. *fumigatus* infections by some unknown mechanisms (40).

### Complement System

In disseminated candidiasis, the activation of complement occurs *via* alternative pathway in a galactomannan/β‐glucan dependent manner. Complement-depleted animals and C5-deficient mice showed increased susceptibility following systemic infection (41, 42). However, no correlation was found between occurrence of disseminated candidiasis and single complement deficiencies in humans (43). Similarly, C5 deficient mice also demonstrated exaggerated susceptibility systemic infection with *A. fumigatus* (44). The protective effect of C5a in Aspergillosis is mainly mediated by its strong chemotactic properties to recruit inflammatory cells in the infected lungs. The complement receptors CR1, CR3 and CR4 on innate cells drive phagocytosis of the opsonized *C*. *neoformans* (45, 46). Additionally, C3a and C5a have been shown to drive the expression of cytokines and chemokines during infection including IL-8 and TNF*α* (47, 48). For *B dermatitidis* infection, glucan deficiency is linked diminished C3‐binding, indicating *β*‐glucan as a critical player of complement activation (49).

### Innate Sensing of the Fungal Pathogens

The interaction between immune cells and fungi is crucial for the generation of functional memory response in IFIs. The recognition of the fungal pathogen associated molecular patterns (PAMPs) by pathogen recognition receptors (PRRs) of the host initiates the antifungal host defense and subsequent development of memory response. The toll-like receptors (TLR; TLR-2, TLR-4, TLR-9), C-type lectin receptors (CLRs; Dectin-1, 2, Galectin, mannose receptor, macrophage-inducible C-type lectin [mincle], dendritic cells specific intercellular adhesion molecule grabbing nonintegrin [DC-SIGN]), nucleotide-binding oligomerization domain (NOD)-like receptors (NLRs) recognize fungal cell wall components such as glucans, mannans, peptidoglycan-associated proteins and phospholipomannan to initiate the antifungal activity (12, 50, 51). The recognition of fungal PAMPs by specific PRRs drives an intracellular signaling cascade in the responding cells that leads to not only the activation of innate and adaptive immune defense but also programs cells to develop and survive as memory cells (52).

### Trained Immunity in Response to IFIs

Innate immune cells can be trained by fungi and fungal components. Recent mouse model studies demonstrated the innate memory response in monocytes/macrophages and NK cells against *C. albicans* (53–55). In this system, mice infected with an attenuated *C. albicans* strain showed protection against invasive Candidiasis (56, 57). The protection is mediated by a T cell-independent but macrophage-dependent manner, popularly known as innate memory response or trained immunity (58). Mechanistically, trained immunity is mediated by epigenetic reprogramming and metabolic shift in innate immune cells (52, 58, 59). Upon recognition of *C. albicans* or β-glucans by monocytes and macrophages, dectin 1-mediated activation of RAF1 signaling cause stable alterations in histone methylation and acetylation. Trained monocytes and macrophages show increased ability to secret cytokines and more efficient and rapid control of fungus following secondary infection (59). Moreover, trained immunity relies on AKT, mammalian target of rapamycin (mTOR) and hypoxia-inducible factor 1α (HIF1α), activation and on a shift of glucose utilization from oxidative phosphorylation to glycolysis (52). Interestingly, BCG and measles vaccinated children showed evidence of memory innate immune response (60, 61). These observations suggest that these vaccines would provide cross-protection to other infections including IFIs (62). Interestingly, defect in trained immunity has also been observed in patients with STAT1 deficiency and suffering from chronic mucocutaneous candidiasis, thus highlighting the clinical importance of this least understood innate defense mechanisms in control of fungal infections (63). Although trained immunity in macrophages and NK cells has been described, the concept of long-term immunological memory development in short-lived neutrophils is difficult to prove.

## Adaptive Immune Components of Functional Antifungal Memory Response

During infection, professional APCs process and present microbial antigen to naive T cells in the context of MHC II. Naïve T cells *via* T cell receptor (TCR) recognize the antigen-MHC II complex and undergo activation and proliferation (17, 64). This event leads to the generation of short-lived effector T cells that clear the pathogen. The activated T cells will also help B cells to produce antibody secreting plasma cells. The generation of short-lived effector T and B cells precedes the development of memory T and B-cells population, respectively. In most cases, the memory T and B cells survive for years, even in the absence of antigens (17, 64). The memory T cells can be divided into two functionally distinct subsets such as T central memory (Tcm) and T effector memory (Tem) cells (65). Upon secondary exposure, the memory T and B cells will respond quickly and clear the pathogen *via* cell and humoral-mediated immunity, respectively.

### CD4 T Cells

Upon antigen encounter, naïve CD4 T cells differentiate into various flavors of Th cells. This include Th1, Th2, Th9, Th17, Th22, regulatory T cells, and follicular helper T cells (**Figure 1**). The most important Th cells in the antifungal immune response against IFIs are the Th1 and Th17 cells (66, 67).

### (i) Th1 Cells

The Th1 response is characterized by the production of pro-inflammatory cytokines such as IFN-γ, which plays important role in host defense against intracellular pathogens. The Th1 cells also offer a protective immune response to the host against IFIs (68). For example, IFN-γ or IFN-γ receptor-deficient mice succumb to invasive Candidiasis and pulmonary Cryptococcosis, respectively (69, 70). In line with the mouse model studies, individuals with invasive Candidiasis showed better prognosis after recombinant IFN-γ therapy (71). IFN-γ therapy also lowered Cryptococcus burden in the cerebrospinal fluid of patients with HIV associated Cryptococcal meningitis (72). Additionally, cell-based therapy with IFN-γ producing *A. fumigatus* specific CD4 T cells protected mice with bonemarrow transplantation and humans following invasive Aspergillosis (73, 74).

### (ii) Th17 Cells

The discovery of Th17 subset has revolutionized our knowledge of the antifungal host defense. APC-derived cytokines like IL-6, IL-1β and IL-23 facilitate Th17 differentiation (75, 76) (**Figure 1**). Th17 cells produce IL-17 family of cytokines (IL-17A-F) that bind to IL-17 receptor subfamilies (IL-17RA-RE) on target cells (77). The target cells of IL-17 are mostly non-hematopoietic cells. Th17 cells *via* the production of IL-17 stimulates the expression of cytokines, chemokines and antimicrobial peptides in target cells, which in turn promote the recruitment of innate cells and clearance of the fungi. Considerable data implicate Th17 cells in immunity to Candida and other fungi (19, 78). Indeed, humans with genetic polymorphisms in IL-17 pathway are at increased risk of fungal infections. For example, mutations in genes controlling IL-17 signaling (*ACT1*, *IL17RA*, *IL17F*), genes that drive Th17 development (*DECTIN1*, *CARD9*, *STAT3*, *STAT1*, *IL12RB*) or in individuals with naturally-occurring anti-IL-17 Abs (AIRE deficiency) showed increased susceptibility to mucosal Candidiasis, but not invasive Candidiasis (79). Thus, the role of IL-17 or Th17 cells in the protection against IFIs remains to be clarified. We and others have shown that IL-17- and IL-17R-deficient mice are highly susceptible to DC (36, 80–82). At mucosal surfaces and skin, IL-17 protects against *C*. *albicans* by inducing the expression of anti-microbial peptides (AMP) that limit infection and chemokines that mediate recruitment of neutrophils with antifungal activities (19, 78). However, we discovered a surprisingly kidney tissue protective role for IL-17 in invasive Candidiasis (81, 82). Supporting these findings, immunization with Als3 containing vaccine conferred protection against invasive Candidiasis by inducing a strong Th1 and Th17 response (83). Additionally, Th1/Th17 paradigm has also been shown to be critical for protection against Cryptococcosis in mice (84).

### CD8 T Cells

The APCs cross-present exogenous fungal antigens to CD8 T cells to activate and differentiate them into Tc1, Tc2, Tc3 subtypes (85). Once activated, CD8 T cells proliferate to become effector cells that control the fungal infection by mediating specific effector functions. These include activating macrophages *via* production of IFN-γ and direct killing of pathogenic fungus. Indeed, mice depleted of CD8 T cells showed increased susceptibility to Cryptococcal infection and CD8 T cells restricted *C. neoformans* growth in infected macrophages by secreting IFN-γ (86, 87). While Tc1 cells secret cytotoxic molecules such as perforin and granzymes to lyse fungal cells, Tc2 cells secrete IL-4 and IL-10 and play important role in immune regulation (88). Interestingly, Tc3 cells secrete IL-17 cytokines and express transcription factor RORγ and CCR6, hallmarks of classical Th17 cells (88). IL-17 produced from Tc3 cells drives the infiltration of innate immune effectors and upregulates the expression of antimicrobial peptides, defensins from the epithelial cells, which are critical for controlling fungal burden in the infected organs (88).

### B Cells

While the most important function of B cells is to produce antibodies during infection, it also contributes to antigen presentation to T cells and cytokine production. During IFIs, antifungal antibodies directed against fungal cell wall components confer host defense (89). Accordingly, passive transfer of serum from vaccinated mice were able to protect against fungal infection (90). Antibodies control fungal agents by multiple mechanisms including preventing fungal entry, activation of classical complement pathway, inhibition of fungal replication, suppression of germ tube formation and inhibiting the formation of fungal biofilms. For example, antibodies against mannoprotein of *C. albicans* showed candidacidal function by suppressing adherence and germination (91). It has also been demonstrated that antibody-mediated iron starvation is an effective way of controlling fungal growth (89). The anti-β-glucans antibodies directly inhibit the growth of *C. albicans* and *C. neoformans* (92). Finally, antibodies against *C. neoformans* capsular antigen interfere with the biofilm formation.

### T and B Cells Memory Response

For antifungal vaccines, it is critical to develop and maintain long-lasting memory response both in immunocompetent and immunocompromised patients. Recently, some of the antifungal vaccine candidates have been tested for various IFIs. These include *C*. *albicans*, *Aspergillus* spp, *Cryptococcus* spp, *Blastomyces* spp, *Paracoccidioides brasiliensis* and *Sporothrix* spp. However, our comprehensive understanding of the memory response in providing long-term protective immunity against IFIs is currently lacking (93).

The various subsets of effector CD4 and CD8 T cells such as Th1, Th17, Tc1 and Tc17 develop into memory cells and confer long term protective immunity (88). A recent study by Nanjappan et al. demonstrated that Tc17 cells persist as long-lasting memory cells by the production of GM-CSF and TNF-α and confers protection against secondary fungal infections (88). Interestingly, Tc17 cells-mediated antifungal resistance was demonstrated in the absence of CD4 T cells, indicating that CD8 T cells can play a dominant role in controlling fungal load in the absence of CD4 T cells (88). This observation is of high clinical relevance since HIV patients who lack CD4-mediated immunity and are at high risk of IFIs (88).

**Figure 2:**
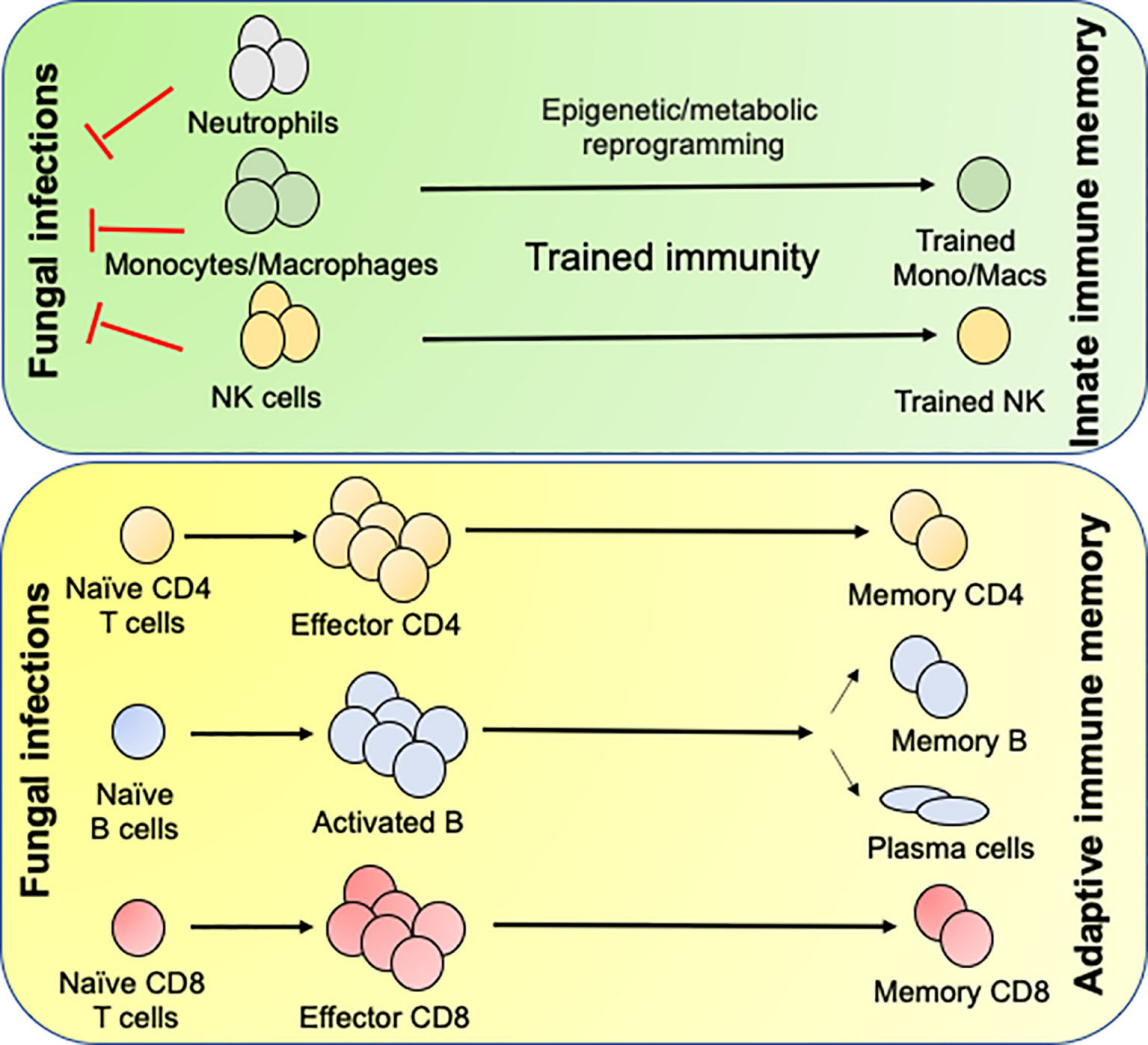
Generation of innate and adaptive antifungal memory response: Following fungal infections, innate cells like neutrophils, monocytes/macrophages and NK cells infiltrate the infected organs and clear the fungi. During this process the monocytes/macrophages and NK cells undergo epigenetic and metabolic reprogramming to form innate memory cells in a process known as Trained immunity. Following initial innate response, naïve CD4 and CD8 T cells recognize fungal antigens in context to APCs and form effector cells, which aid in the clearance of infection. Once the infection is cleared most of the effector cells die leaving few CD4 and CD8 T cells to become long lived memory cells. The memory CD4 and CD8 T cells either reside in the secondary lymphoid organs or tissues and confer rapid protection following secondary fungal infections. B cells recognize antigen by the B cell receptor and signals derived from the antigen specific CD4 T cells in the T/B zone of the secondary lymphoid organs. Some activated B cells form short-lived plasmablasts while others enter germinal center (GC). In the GC, the B cells undergo clonal expansion, somatic hypermutation and class-switch recombination. Antigen selected B cells eventually differentiate into memory B cells or antibody secreting long lived plasma cells, which migrate to the bone marrow.

The formation of germinal centers in the secondary lymphoid organs precedes the formation of memory B cells (MBCs) following primary infection (**Figure 2**). Once formed, the MBCs survive for long time and repeatedly produce antibody in the case of re-infections. Earlier studies from the mouse models elucidated that many therapeutic vaccines mediate antifungal protection by generating the antibody (89, 91, 94). Interestingly, some of the anti-β-glucan antibodies cross-react with several other fungal pathogens, indicating that single antibody may protect against multiple fungal pathogens (92). In contrast, antibodies generated during primary IFIs and naturally acquired antibodies at the stage of early childhood infection was unable to protect against subsequent IFIs (95). This data indicates that anti-fungus antibodies is not sufficient to prevent future fungal infections and T cell-mediated immune response plays a significant role in the long-term.

## Efforts to Develop New Antifungal Vaccines

**Figure 3:**
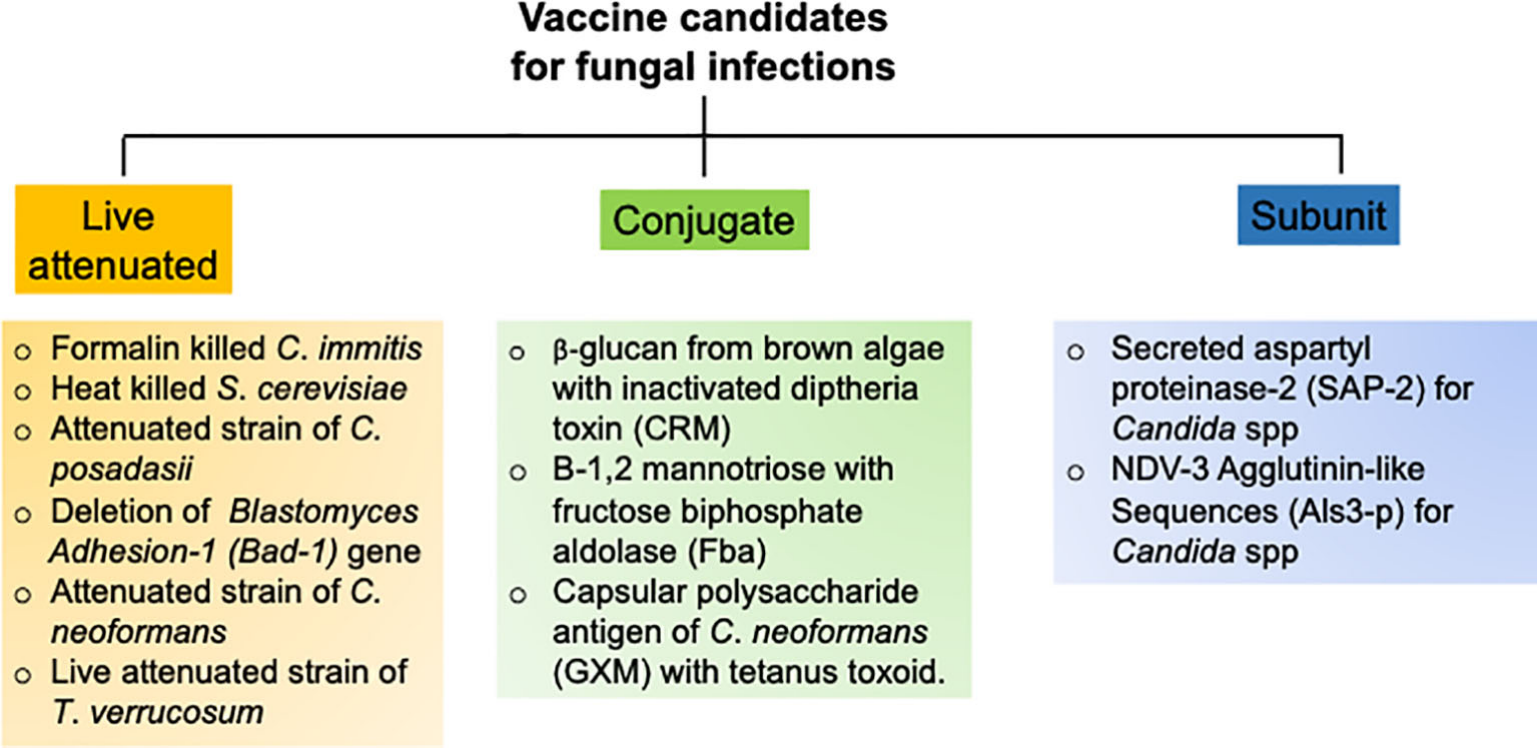
Research strategies to develop successful antifungal vaccines.

The goal of an efficient fungal vaccine is to generate long-lasting immunological memory and provide sterile immunity against a recurrent exposure to the fungi (93). To achieve these objectives, several vaccination strategies with different vaccine candidates have been tested in pre-clinical animal models and clinical trials, which are outlined below (**Figure 3**).

### C. albicans

Over the past decade, numerous groups have assessed multiple candidate vaccines to develop sterilizing and long-term antifungal immunity against *C*. *albicans*. These candidates include polysaccharides from fungal cell wall, fungal protein subunits and live attenuated strains of *C. albicans* (96). The most promising vaccine strategy ever tested is the use of N-terminal portion of the agglutinin like sequence 3 protein (Als3p) of *C*. *albicans* (97). Mice and non-human primates were immunized with purified Alsp3 protein mixed with aluminum hydroxide as adjuvant. Following encouraging results in animal models, the vaccine was further subjected to clinical trials in human. Seventy-three adults were vaccinated with two doses and placebo as control. All the subjects generated robust anti-Als3p IgG antibody after the first dose. The second dose elicited a strong IgA1 antibody titer in addition to IgG response. The vaccine successfully elicited T-cell response, as evident by the production of T cells cytokine such as IL-17 and IFN-γ, which were critical for anti-Candida immunity. One of the major limitations of this study is that it has not been tested in individuals receiving corticosteroids and antibiotics. In a separate study, a recombinant version of the secreted aspartyl proteinase 2 (Sap-2) protein of *C*. *albicans* was used to vaccinate mice and assess susceptibility to vaginitis (98). The Sap-2 protein along with virosome as adjuvant were vaccinated by intravaginal route. The vaccination resulted in the generation of Sap-2 specific protective antibodies, which also cross reacted with different Saps (99). Additionally, a live attenuated strain of *C. albicans* and has also been tested in the mouse models (98–100). Although these vaccine strategies showed promising results, they have not been tested in humans (98). This is primarily due to the high risk of introducing attenuated live fungus in humans particularly in the immunocompromised individuals.

### *Aspergillus* spp

The pulmonary Aspergillosis is a serious infection in immunocompromised patients. However, it is also observed in immunocompetent individuals, indicating that a vaccine against *Aspergillus* spp should be capable of inducing protective immunity in both immunosuppressed and immunocompetent subjects. A study by Cenci et al. showed that intranasal immunization of crude *Aspergillus* antigens was effective in generating Th1 immunity and protected mice from pulmonary aspergillosis (73). Notably, a follow-up study using sonicated fungal antigen was successful to generate protective immunity in corticosteroid treated immunosuppressed mice. This data indicates that it is possible to protect against pulmonary Aspergillosis in the face of immunosuppression, which is a major clinical problem in hospital settings (101). In a separate report, epitope p41 from the cell wall glucanase of *A. fumigatus* was used to vaccinate mice against Aspergillosis (102). The antigenic epitope was presented by professional APCs through to generate Th1 response. Interestingly, the antifungal CD4 T cells response generated by p41 epitope can cross-react with *C. albicans*. Additionally, a pan-fungal vaccine using β-glucans of *S. cerevisiae* generated optimal protection against several pathogenic fungi, including *A. fumigatus* (103). This vaccine can generate protective immunity in the absence of adjuvants, which is an added advantage. Since, this study was performed in immunocompetent animals, it is not known whether this immunization strategy will work similarly in immunocompromised individuals. Nevertheless, the idea of a pan-fungal vaccine to protect against these multiple deadly fungal diseases is interesting and warrants careful consideration in the near future (103, 104).

### *Cryptococcus* spp

Cryptococcosis is the most common cause of HIV-related meningitis. Hence, vaccines against *Cryptococcus* spp need to be efficient in the absence of functional CD4 T cells, which are primary cells in helping B cells to produce antibodies (8). Following an asymptomatic acute phase infection, the fungus enters into latent state. In the face of immunosuppression, the latent fungus reactivates to cause disease in HIV infected individuals (105). Hence, a successful vaccine should be able to restrain both the acute and recurrent infection (105). The first vaccine designed to immunize mice against *C. neoformans* comprised of an antiphagocytic antigen from the capsule of *C. neoformans*, known as glucuronoxylomannann (GMX) (106). Importantly, GMX does not require T cell help for the generation of antibody response. To enhance the antigenicity of the vaccine, GMX was mixed with tetanus toxoid and injected in mice (106). Although vaccinated mice developed strong anti-Cryptococcus-specific antibody response, most of these antibodies are non-protective (105, 107). A separate group vaccinated T cells depleted mice with an strain of *C. neoformans* that were engineered to express IFN-γ (108). Vaccination protected the T cells deficient mice following a secondary pulmonary infection using a pathogenic strain. This data for the first time showed the feasibility of mounting a protective immune response to *Cryptococcus* in the absence of normal immune system. Subsequently, a study utilized a live attenuated strain to immunize mice and assess protection against virulent *Cryptococcus* strains (109). In this setting, the mutant lacked the sterol glucosidase enzyme (*Δsgl1*), leading to an increased production of sterol glucosides in the cells. Infection with the *Δsgl1* fungus enable mice withstand a secondary infection with virulent strains of *C. neoformans* and *C. gattii*.

### Endemic Mycoses

Mice depleted of CD4 T cells and immunized with an attenuated strain of *B. dermatitidis* (lacking the gene for the adhesin Bad-1) showed protection against the infection by a wild type virulent strain of *B. dermatitidis* (110). This observation highlights the importance of CD4 T cell independent immune pathways to generate protective immunity against endemic blastmycosis. Another interesting study showed that immunization with P10, a modified peptide derived from the antigen gp43 of *P. brasiliensis*, protected both immunocompetent and immunocompromised mice against this pathogen (111). Moreover, immunization with *S. cerevisiae* expressing gp43 conferred resistance in mice, as evident by lower fungal burden and production of IL-12 and IFN-γ in the lungs (112). Finally, de Almeida et al. showed that treatment of mice with antibodies against the glycoprotein 70 (gp70) from *S. schenkii* showed protection against infection with different strains of *S. schenkii* and *S. brasiliensis* (113). This result suggest that passive antibody therapies can be a treatment option in immunocompromised patients against endemic mycoses (96).

## Conclusions

The biomedical science has been blessed with many successful vaccines that have prevented millions of deaths and irradicated many infectious diseases. Even now, there are no approved vaccines against any fungal pathogens and the IFIs continue to be a major threat to human health especially among the immunocompromised patients. Limitations associated with conventional antifungal diagnosis and drugs further aggravated the scenario. Extensive research over the past decades have shown that vaccination against one fungus can confer protection against the other, thus laying the groundwork for the development and validation of universal antifungal vaccine concept. Thus, the research for the generation of universal fungal vaccines have gained momentum and popularity in the research community, which may pave the path for the development of fungal vaccines against wide array of fungal pathogens. These efforts may not only prove beneficial in discovering fungal vaccine candidates from unexpected and distant species, but also may provide information on re-purposing some of the successful vaccines used against other pathogens in fungal diseases.

## Author Contributions

The author confirms being the sole contributor of this work and has approved it for publication.

## Funding

This work was supported by a Rheumatology Research Foundation grant to PB and NIH grants AI145242, AI142354 and DK104680 to PB.

## Conflict of Interest

The author declares that the research was conducted in the absence of any commercial or financial relationships that could be construed as a potential conflict of interest.

The handling editor declared a past co-authorship with the author.
